# Effect of Surfactants and Manufacturing Methods on the Electrical and Thermal Conductivity of Carbon Nanotube/Silicone Composites

**DOI:** 10.3390/molecules171113157

**Published:** 2012-11-05

**Authors:** Jarmila Vilčáková, Robert Moučka, Petr Svoboda, Markéta Ilčíková, Natalia Kazantseva, Martina Hřibová, Matej Mičušík, Mária Omastová

**Affiliations:** 1 Polymer Centre, Faculty of Technology, Tomas Bata University in Zlín, Zlín 760 01, Czech Republic; 2 Centre of Polymer Systems, University Institute, Tomas Bata University in Zlín, Nad Ovcirnou, Zlín 760 01, Czech Republic; 3 Polymer Institute, Slovak Academy of Sciences, Bratislava 845 41, Slovak

**Keywords:** multi-wall carbon nanotubes, modification of carbon nanotubes by ionic surfactants, silicone based composites, electrical conductivity, thermal conductivity, percolation threshold, complex permittivity

## Abstract

The effect of ionic surfactants and manufacturing methods on the separation and distribution of multi-wall carbon nanotubes (CNTs) in a silicone matrix are investigated. The CNTs are dispersed in an aqueous solution of the anionic surfactant dodecylbenzene sulfonic acid (DBSA), the cationic surfactant cetyltrimethylammonium bromide (CTAB), and in a DBSA/CTAB surfactant mixture. Four types of CNT-based composites of various concentrations from 0 to 6 vol.% are prepared by simple mechanical mixing and sonication. The morphology, electrical and thermal conductivity of the CNT-based composites are analyzed. The incorporation of both neat and modified CNTs leads to an increase in electrical and thermal conductivity. The dependence of DC conductivity *versus* CNT concentration shows percolation behaviour with a percolation threshold of about 2 vol.% in composites with neat CNT. The modification of CNTs by DBSA increases the percolation threshold to 4 vol.% due to the isolation/separation of individual CNTs. This, in turn, results in a significant decrease in the complex permittivity of CNT–DBSA-based composites. In contrast to the percolation behaviour of DC conductivity, the concentration dependence of thermal conductivity exhibits a linear dependence, the thermal conductivity of composites with modified CNTs being lower than that of composites with neat CNTs. All these results provide evidence that the modification of CNTs by DBSA followed by sonication allows one to produce composites with high homogeneity.

## 1. Introduction

Carbon nanotubes (CNTs) are of great interest as a conductive filler for polymer composites. Owing to their potential mechanical, electrical and thermal properties, CNTs have attracted considerable scientific attention and have been the subject of numerous studies since their development [1,2]. The method of CNT synthesis determines the surface electronic structure of the nanotubes and their conducting properties. During their synthesis, CNTs are strongly entangled, forming aggregates due to van der Waals forces [3]. To create polymer nanocomposites with CNTs, one should uniformly disperse CNTs over a polymer matrix, while maintaining the aspect ratio and the electronic structure of the CNTs [4]. To solve this problem, several methods of de-agglomeration of CNTs have been developed. Basically, these methods can be divided into mechanical and chemical methods [5]. In turn, the chemical methods can be divided into covalent and non-covalent ones. In the former method, CNTs are separated due to the formation of covalent bonds between various chemical groups and the CNT surface. In the non-covalent method, CNTs are de-aggregated due to van der Waals forces between the CNT surface and modifiers [6,7], for example, ionic surfactants [8].

Surfactants are classified according to the sign of the charge on a surfactant molecule when it is dissolved in water. Generally, there are two groups of surfactants–non-ionic, with no charge in its head, and ionic: cationic, anionic and zwitterionic. Ionic surfactants can be used with water soluble polymers such as polyvinyl alcohol (PVA) [9]. In our work we used an anionic surfactant–dodecylbenzenesulfonic acid (DBSA)—which forms a negative charge when dissolved in water, a cationic surfactant—cetyltrimethylammonium bromide (CTAB)—forming a positive charge when dissolved in water, and a DBSA/CTAB surfactant mixture, with the aim of studying the influence of surfactant type on the dispersion in solution of CNTs. Surfactant can also cause the non-covalent modification of the CNT surface, which is helpful for dispersion of this kind of filler in polymeric matrix.

The high electrical conductivity of CNTs makes them good filler candidates for electrically conductive polymer composites. For a certain critical concentration of a conducting filler, the so-called percolation threshold *φ*_c_ [10], the conductivity of a composite exhibits a transition from dielectric to conducting state. The value of *φ*_c_ depends on the shape and the aspect ratio of the filler, the production process, and the type of interaction between the filler and the polymer matrix. Experimental values of *φ*_c_ in CNT-based composites vary significantly (from 0.1 to 10 wt.%) [11,12]. A composite of polycarbonate (PC) with CNTs produced by melt mixing using the masterbatch dilution method has *φ*_c_ ~1.0 wt.% [13]. Polymethyl methacrylate (PMMA) composites with CNTs prepared by solvent casting technique demonstrate *φ*_c_ of about 4 wt.% [14]. Polyethylene (PE)–CNT composites prepared by melt blending using a mini-twin screw extruder show a *φ*_c_ of about 7.5 wt.% [15]. The percolation threshold of polypropylene (PP)–CNT nanocomposites prepared by diluting a master-batch with different types of PPs varied from 1.1 to 2.0 vol.%; the lower values were obtained for matrices with high melt flow index [16]. In our research we used polydimethylsiloxane (PDMS) as a matrix due to its flexibility and ease of processing. Composites of PDMS with CNTs are important for creating new electronic devices [17].

To date much work has been done in obtaining CNT–silicone composites after chemical pre-treatment of CNTs by silane coupling agents. Vast [18] introduced CNTs modified by 7-octenyl-trichlorosilane and showed that oxidation of CNTs induces defects, which does not allow one to obtain conducting composites. Chen *et al*. [19] showed that the addition of up to 5 wt.% of CNTs modified by 3-aminopropyltriethoxysilane to a silicone matrix did not change the electrical conductivity of the composite. Chua *et al*. [20] observed an increase of electrical conductivity of silicone composites from 10^−7^ to 10^−4^ S·m^−1^ in the range from 0.5 to 2 wt.% of modified CNTs in the same silane. The electrical conductivity of unmodified CNT/PDMS nanocomposites prepared by soft lithography micromolding increased from 10^−3^ to 10^−2^ S·m^−1^ (from 0.5 to 2.5 wt.%) as reported by Khosla and Gray [21]. Hwang *et al*. [22] achieved a homogeneous dispersion of CNTs in PDMS using a wrapping method for CNT modification with poly(3-hexylthiophene). However the value of electrical conductivity of the resulting nanocomposites was found to be low.

An important property of CNTs is their high thermal conductivity, which is about 3,000 W·m^−1^K^−1^ in multi-walled CNTs and ranges from 2,000 to 6,000 W·m^−1^K^−1^ in single-walled CNTs. This makes CNTs one of the most promising fillers for the design of thermally conductive composites [23]. However, in practice the thermal conductivity of CNT/polymer nanocomposites is much lower compared with the conductivity of neat CNTs because of the interfacial thermal resistance between CNTs and the polymer matrix [24].

The present work is devoted to the development of homogeneous silicone composites filled with CNTs. To this end, we modify CNTs with an ionic surfactant, which provides uniform distribution of CNTs in a polymer but does not affect the electronic structure of the CNTs. We study the effect of surfactants followed by sonication on the morphology and the electrical and thermal conductivity of CNT-based composites. To this end, we prepare three types of CNT/silicone composites with filler concentrations varying from 0 to 6 vol.% and compare the electrical and thermal conductivity of the composites with modified and neat CNTs.

## 2. Results and Discussion

### 2.1. Morphology of Modified and Neat CNTs: Effect of Ionic Surfactants

**Figure 1:**
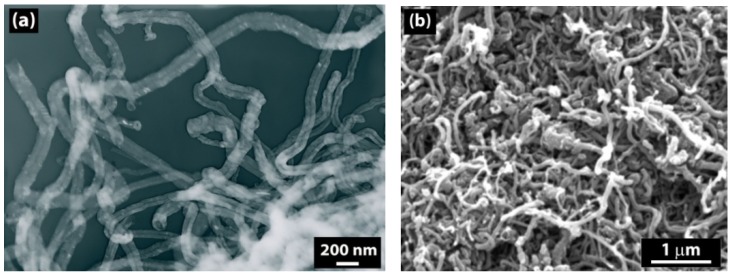
(**a**) TEM and (**b**) SEM images of neat CNTs.

**Figure 2:**
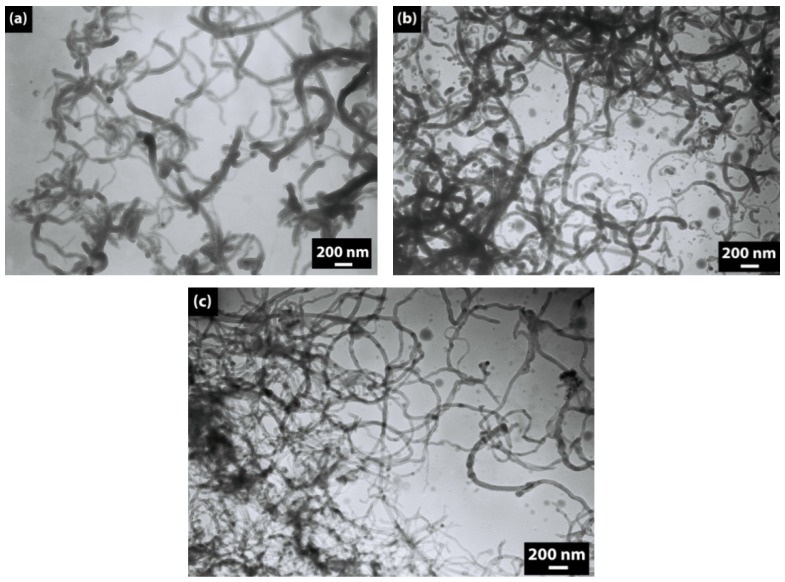
TEM micrographs of (**a**) CNT–DBSA, (**b**) CNT–CTAB, and (**c**) CNT–DBSA–CTAB.

The morphology of neat and modified CNTs is shown in Figure 1 and Figure 2. According to the TEM images, the diameter of the neat CNTs is about 30–40 nm, and the SEM images show that CNTs create aggregates. 

Modification of CNTs by CTAB and by a DBSA–CTAB mixture does not produce a desired effect of separation of CNTs (Figure 2b,c). In contrast, the use of DBSA followed by sonication leads to a proper separation of CNTs (Figure 2a). The positive effect of anionic surfactant on the disentanglement of CNTs can be explained in the following way: it is known that the charged head group of an ionic surfactant plays an important role in the location of the surfactant on the CNT surface [25,26]. In the case of an anionic surfactant (DBSA), the hydrophobic interaction between alkyl chains of DBSA and CNTs surface is likely to play the main role in the isolation/separation of CNTs. On the other hand, in the case of a cationic surfactant (CTAB), as well as of a mixture of cationic and anionic surfactants (DBSA–CTAB), a micellar structure is formed.

### 2.2. Thermogravimetric Analysis of Neat CNTs and CNTs Modified by DBSA

**Figure 3:**
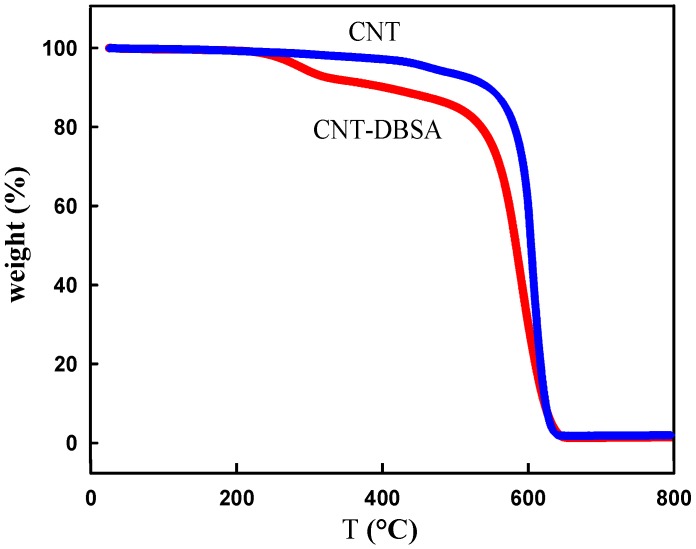
TGA curves of CNTs and CNTs modified by DBSA.

Thermogravimetric analysis (TGA) was used to determine the mass loading of surfactants modifiers. Weight loss of CNT starts at 550 °C and the maximum decomposition temperature is 647 °C, as obtained from derivation of TGA curve (not shown in Figure 3). The residue of about 1.5 wt.% comes from catalysts, which corresponds with the given purity of used nanotubes. TGA curves of CNT modified with the anionic surfactant DBSA, evidently show two decomposition steps. The first one, at lower temperature, corresponds to the decomposition of the surfactants and the second one at about 600 °C is assigned to the decomposition of CNT (Figure 3). The CNT–DBSA sample has a decomposition maximum at 256 °C and the amount of surfactant present in the sample is about 8 wt.%. Sample was modified using theoretically 20 wt.% of surfactants to the mass of CNTs, but the real surfactant content in the modified CNT is lower. This is partially caused by the filtration procedure when a part of the surfactant was washed out. 

### 2.3. Morphology of CNT based Composites

**Figure 4:**
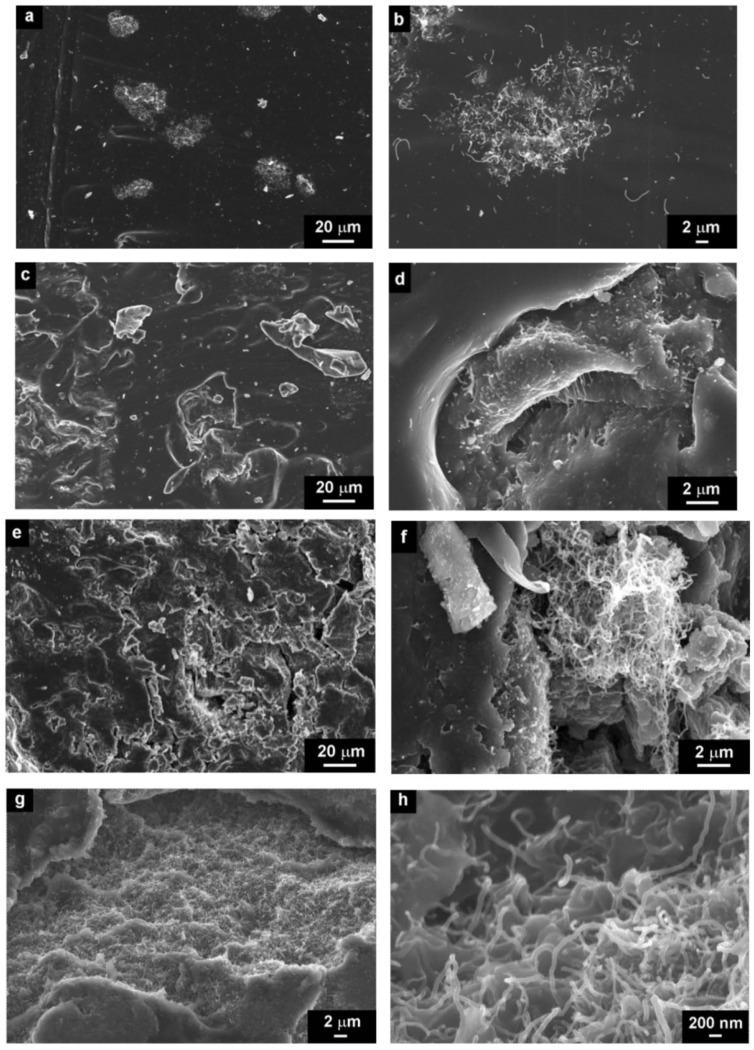
SEM micrographs of CNT/silicone composites with different concentrations (**a**,**b**) 0.5 vol.% (**c**,**d**) 1.5 vol.%, (**e**,**f**) 3.5 vol.%, (**g**,**h**) 5 vol.%, prepared by mechanical mixing.

**Figure 5:**
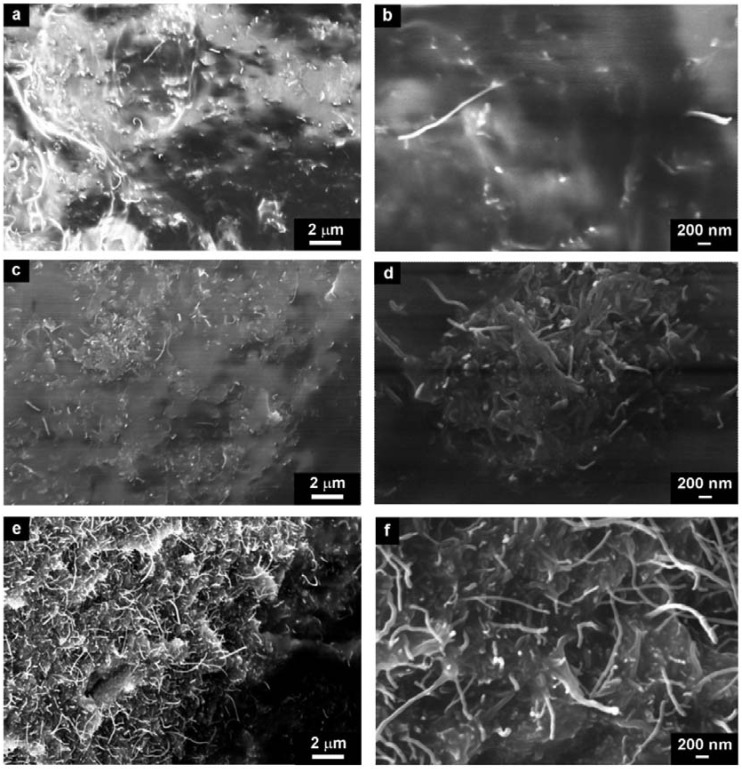
SEM micrographs of CNT–DBSA/silicone composites with different concentrations (**a**,**b**) 2,5 vol.%, (**c**,**d**) 3.4 vol.% (**e**,**f**) 4.2 vol.%, prepared by sonication.

We prepared two sets of CNT composites of different volume filler concentrations. Both sets were prepared by two different processing conditions: mechanical mixing and by sonication. In the first set, we used neat CNTs (Figure 4) as a filler and in the second set, we used CNTs modified by anionic surfactant (DBSA) (Figure 5).

Four concentration regions were chosen to study the morphology of CNT/silicone-composites: 0.5 vol.% of CNT, which is below *φ_c_* (Figure 4a,b), 1.5 vol.% of CNT, which is at the *φ*_c_ (Figure 4c,d), 3.5 vol.% of CNT (Figure 4e,f) and 5 vol.% of CNT, which is above *φ_c_* (Figure 4g,h).

The micrographs show the structures of composites below, at and above the critical CNT concentration at different magnification scales. According to the results obtained, there are large aggregates of CNTs, even at low CNT concentrations in the silicone matrix. The amount of aggregates increases with the CNT concentration, which in turn leads to the defect structure of composites (Figure 4d,f,g).

The structure of CNT–DBSA/silicone composite were investigated in three concentration regions, namely below *φ_c_* (2.5 vol.% of CNT–DBSA, Figure 5a,b), at the *φ*_c_ (3.4 vol.% CNT–DBSA, Figure 5c,d), and above the *φ*_c_ (4.2 vol.% CNT–DBSA, Figure 5e,f).

In contrast with CNTs-based composites, CNT–DBSA/silicone composites do not show the presence of CNT aggregates at the whole concentration region, even above *φ*_c_. It is visible from the SEM images that the distribution of CNT–DBSA in silicone is uniform. Moreover, the surface of CNT–DBSA/silicone composite is smooth, which indicate the presence of filler-matrix interaction, resulting in denseness of composites.

### 2.4. Correlation between the Morphology and the Electrical Properties of CNT-Based Composites DC Conductivity

**Figure 6:**
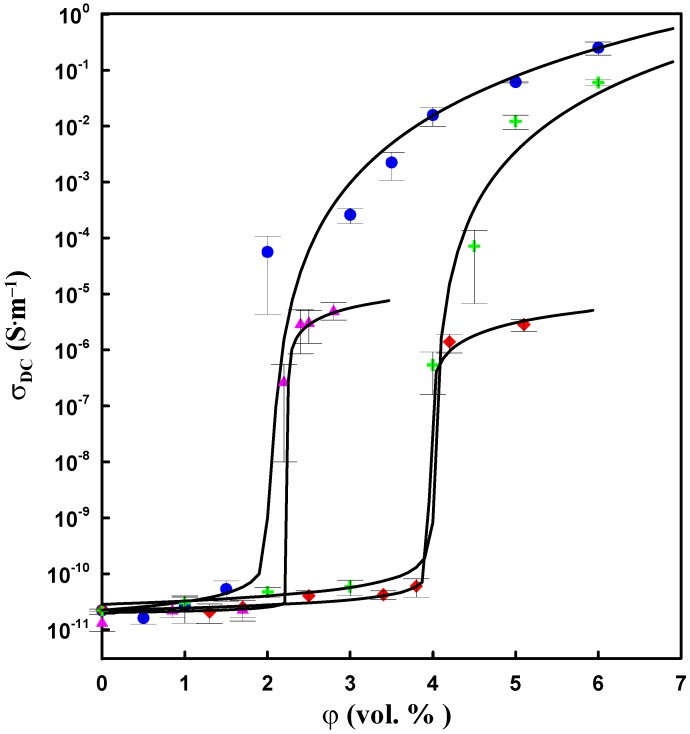
The dependence of DC electrical conductivity on CNT content is shown for CNT/silicone composite (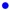 mechanical mixing, 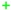 sonication) and CNT–DBSA/silicone composites (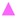 mechanical mixing, 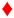 sonication).

The incorporation of neat and modified CNT increases the electrical conductivity of composites (Figure 6). The dependence of DC conductivity *versus* CNT concentration demonstrates a percolation behaviour. The experimental data were fitted by the model proposed by Kirkpatrick [10]. According to this model, for a filler content below *φ*_c_, the electrical conductivity follows the power law:

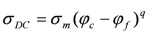
(1)
where *σ_DC_* is the electrical conductivity of composite, *σ_m_* is the electrical conductivity of the matrix, *φ_c_* is the critical filler volume fraction, *φ_f_* is the filler volume fraction, and *q* is an experimentally determined exponent.

In accordance with percolation theory, the electrical conductivity of a composite increases considerably as infinite conductive clusters are formed in the composite. The composites studied differ in *φ*_c_ and the value of electric conductivity above the percolation threshold. Both sonication and filler modification increase *φ*_c_. Thus, the highest value of *φ*_c_ (~4 vol.%) was obtained when CNTs are modified by DBSA and dispersed by sonication. The composites prepared by mechanical mixing of neat CNTs with silicone show the lowest value of *φ*_c_ (~2 vol.%). Therefore, the homogenization of CNTs in a silicone matrix, *i.e.*, the separation of nanotubes, decreases the probability of formation of conductive clusters and thus leads to higher values of *φ*_c_. The variation of *φ*_c_ in CNT-based composites correlates with the morphology of the composites. The modification of CNTs by DBSA followed by sonication leads to the separation of CNTs (Figure 5e) and thus decreases the *φ*_c_ compared with that of composites with neat CNTs, which contain CNT agglomerates (Figure 4f). 

### 2.5. AC Conductivity

**Figure 7:**
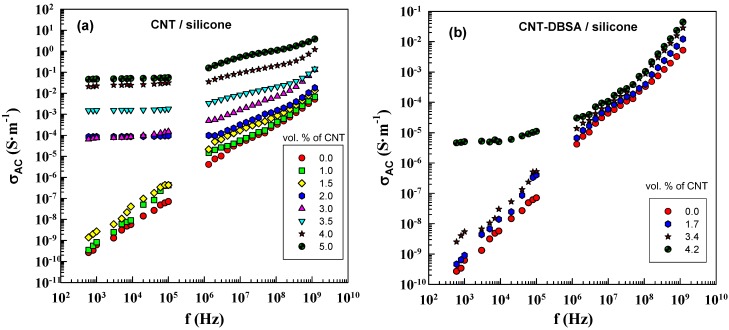
AC electrical conductivity as a function of frequency for (**a**) CNT/silicone (mechanical mixing) and (**b**) CNT–DBSA/silicone composites (sonication) with various filler contents.

The frequency dependence of AC conductivity, *σ_AC_* of CNT/silicone and CNT–DBSA/silicone composites at different filler concentrations is illustrated in Figure 7. According to Equation (6), *σ_AC_* is proportional to frequency and the imaginary part of the complex permittivity. The value of the imaginary part of complex permittivity is determined by the filler content. 

The DC conductivity manifests itself in the frequency dependence of AC conductivity in the form of a plateau region. This is in agreement with the fact that the DC plateau appears above *φ*_c_. Composites with filler concentrations lower than *φ*_c_ do not have conductive paths; in this case, conductivity is due to polarization processes. Composites with filler concentrations above *φ*_c_ (2 vol.% for CNT/silicone composite) exhibit a DC plateau. In the case of CNT–DBSA, the DC plateau appears only at 4 vol.% of the filler, which is in good agreement with the results of DC conductivity measurements.

### 2.6. Dielectric Properties

**Figure 8:**
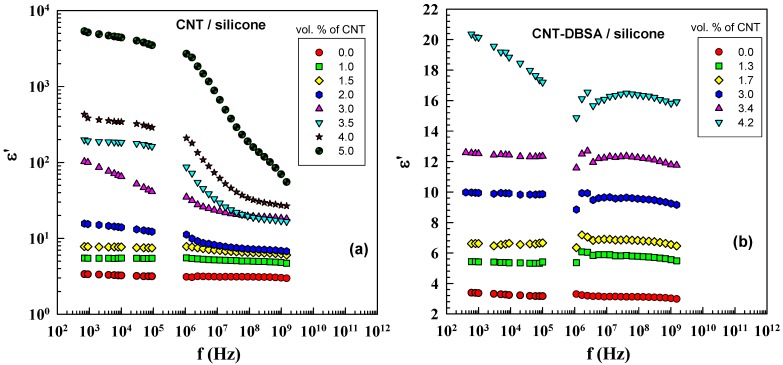
Real part of complex permittivity as a function of frequency for (**a**) CNT/silicone composites (mechanical mixing) and (**b**) CNT–DBSA/silicone composites (sonication) with various filler contents.

The dielectric properties of CNT-based composites were examined in an alternating electric field. Figure 8 shows the real part of complex permittivity of CNT/silicone and CNT–DBSA/silicone composites. The results obtained are in agreement with assumption that the number of charge carriers in composites increases with the filler concentration. It is well known that, at low frequencies, all types of polarization contribute to the resulting value of permittivity; the dominant contribution is made by interfacial polarization, which is typical of materials consisting of phases with different conductivities. At high frequencies, the permittivity is determined by the atomic and electronic polarizations; therefore, there is little difference between the composites. 

The difference in *ε'* between the CNT/silicone and CNT–DBSA/silicone composites increases rapidly as the filler concentration increases. Both composites do not differ significantly at 1.5 vol.% of filler: *ε'* is 8 for CNT/silicone and at 1.7 vol.% filler: *ε'* is 7 for CNT–DBSA/silicone. However, at 4 vol.% of filler (above *φ*_c_) *ε'* of CNT/silicone is about 2,500, whereas, in CNT–DBSA/silicone, *ε'* = 20.

**Figure 9:**
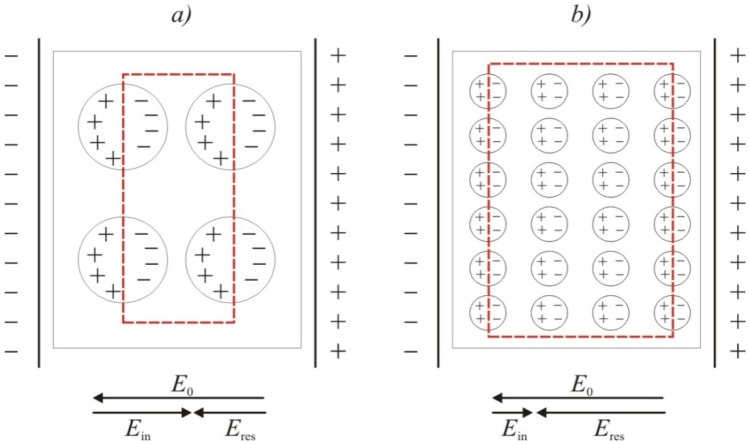
Schematic illustration of interfacial polarization of composites: (**a**) poor dispersion (large agglomerates); (**b**) good dispersion (small or no agglomerates).

Since both composites contain the same amount of filler, this enormous difference between their permittivity *ε'* is likely to be attributed to the composite structure (see the schematic illustration of interfacial polarization in Figure 9). 

The systems of well (modified) and poorly (neat) dispersed CNTs do not differ significantly in the number of charge carriers. However, neat CNTs form large bundles (represented as bigger circlets in Figure 9a) whose count is much lower compared to much less aggregated modified CNTs with many but small bundles (Figure 9b). The intensity of electric field in the system (*E*_in_) with large clusters is higher due to stronger effect of charge elimination (see Figure 9). Thus also its permittivity, *i.e.*, the ratio of *E*_0_ to *E*_res_, is proportionally higher.

### 2.7. Thermal Conductivity

**Figure 10:**
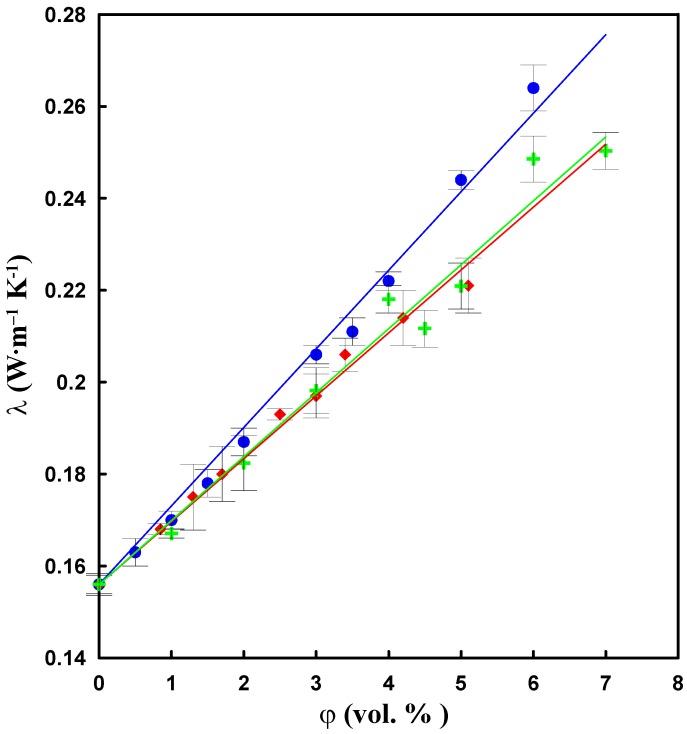
The dependence of thermal conductivity on the CNT content for CNT/silicone composite (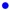 mechanical mixing, 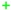 sonication) and CNT–DBSA/silicone composites (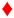 sonication). Experimental data fitted by linear regression.

The thermal conductivity of composites is shown in Figure 10 as a function of CNT concentration. 

Incorporating of CNTs to the silicone matrix significantly increases the thermal conductivity of the composite: while the thermal conductivity of pure silicone is 0.16 W·m^−1^K^−1^, the thermal conductivity of a composite with 6 vol.% of CNT is 0.27 W·m^−1^K^−1^. 

The considerable difference in the morphology of composites (Figure 4) also manifests itself in the thermal conductivity (*λ*). In contrast to electrical conductivity, which exhibits a percolative behaviour due to the presence of conductive paths formed by the filler, the behaviour of thermal conductivity is determined by the large interfacial thermal resistance between the filler and the matrix [27]. Even though some authors [4] employ Lichtenecker’s equation [28] to describe the concentration dependence of thermal conductivity of CNT-based composites, often experimental results are quite well approximated by a linear model [29]. This also applies to our case.

Despite the fact that all three investigated system (CNT/mechanical mixing, CNT/sonication and CNT–DBSA/sonication in silicone matrix) show a linear dependence on filler content, the slopes of *λ vs.* concentration differ significantly. Considering that in all cases we deal with the same filler concentration, this implies that the resulting *λ* depends on how well the filler is dispersed in the matrix. Assuming that the reduction in the heat transfer is caused by phonon scattering on the filler/matrix interface, one can expect that highest reduction occurs in the case of the best homogenisation, which has largest area of filler/matrix interface (the case of CNT–DBSA/sonication).

Thus, the concentration dependence of thermal conductivity in the case of CNT–DBSA can be explained in the following way. At low concentrations of filler, the intrinsic thermal conductivity of filler plays a dominant role in increasing the thermal conductivity of the composites; however, as the concentration of filler increases, the dominant role moves to the resistance of the overall contact region [30].

## 3. Experimental

### 3.1. Materials

**Figure 11:**
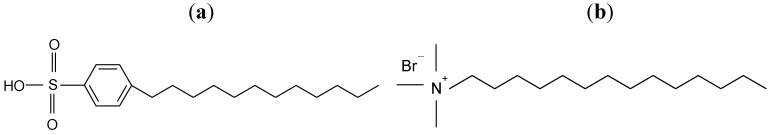
Chemical structure of (**a**) DBSA and (**b**) CTAB.

As filler, we used commercially available multiwall CNTs (MWNT–2040, Conyuan Biochemical Technology, Taipei, Taiwan). The fundamental properties of nanotubes are as follows: purity ≥ 95%, diameter of 20–40 nm, length of 5–15 µm, specific surface area of 40–300 m^2^·g^−1^, and density of 1.8 g·cm^−1^ according to the authors of [31]. We chose Sylgard 184 silicone elastomer (Dow Corning, Midland, MI, USA) as a polymer matrix for its thermal stability (up to 200 °C), resistance to oxygen and flexibility. The silicone elastomer was supplied in the liquid form and consisted of Part A (Base) and Part B (Curing agent). To modify CNTs, we used DBSA (Figure 11a) with purity >90% (Sigma-Aldrich, Sant Louis, MO, USA) as an anionic surfactant and CTAB (Figure 11b) with purity >98% (Penta, Prague, Czech Republic) as a cationic surfactant. Acetone (99.5%, Mikrochem, Pezinok, Slovakia) and distilled water were used without further purification. 

### 3.2. CNT Modification

For non-covalent surface modification of CNTs, we used anionic surfactant (DBSA), cationic surfactant (CTAB) and a mixture of DBSA and CTAB (with molar ratio of 1:1). The rate of CNT entanglement was evaluated by scanning electron microscope images.

The calculated weight fraction of a surfactant (DBSA, CTAB, or a mixture of surfactants) was stirred in distilled water (200 mL) for 15 min. Subsequently, CNTs (2 g) in distilled water (200 mL) was added and the mixture was stirred for 50 min. The amount of surfactants was 20 wt.% of the weight of the final composite. After that we subjected the product to ultrasonic treatment for 50 min using a Hielscher 400S ultrasound finger (Hielscher, Teltow, Germany). Then the suspension (covered by perforated aluminum foil) was kept for 14 h. Next, we filtered the product through filter paper with glass fibres and porosity of 0.2 µm (Millipore) and rinsed several times in distilled water. Finally, the samples were dried at 60 °C and atmospheric pressure for 14 h. The product was carefully ground by pestle in a mortar. We prepared three sets of composites with CNT volume varying from 0 to 6 vol.%. In all cases, we used silicone as a polymer matrix.

### 3.3. Thermogravimetric Analysis

Thermogravimetric analysis (TGA) was performed on samples of CNTs and CNT DBSA about 7 mg by weight in air flow (50 mL·min^−1^). The heating rate was set at 10 °C·min^−1^ over temperature range from 25 °C to 800 °C by using a thermogravimeter (SETARAM TG-GA 12, Thermal Analysis Instruments, New Castle, DE, USA). The results were evaluated with the TA Universal Analysis programme.

### 3.4. Preparation of Neat CNT/Silicone Composites by Mechanical Mixing

The first set of composites consisted of several samples with CNT concentrations varying from 0 to 6 vol.%. We placed the calculated amount of neat CNT and silicone in a 50-mL beaker and mixed them by an EURO-ST-D mechanical stirrer (IKA Labortechnik, Staufen, Germany) for 30 min; then we added a catalyst and mixed the product another 30 min. 1-mm-thick disc-shaped samples, 15 mm in diameter, were produced by cast moulding into vacuum desiccators, where air bubbles were removed. Finally, the form was closed and placed into a drying oven, where the material was cured at 100 °C for 2 h. 

### 3.5. Preparation of Neat CNT/Silicone Composites by Sonication

The second set of composites with neat CNTs consisted of samples with filler concentration varying from 0 to 6 vol.%. We placed the calculated amount of neat CNT and 20 mL of acetone into a 50-mL beaker and sonicated for 60 min by an UP 400s ultraprobe at frequency of 24 kHz and power of 400 W. After that, we added a matrix and continued the sonication for another 5 min. Then the beaker with mixture was placed into a dish with hot oil (100 °C) and mixed by a magnetic stirrer until all acetone was evaporated. After that we added a catalyst, mixed the components by a glass stick for 5 min, and then cast into vacuum desiccators in order to remove air bubbles from the material. 1-mm-thick disc-shaped samples, 15 mm in diameter, were produced by cast moulding into vacuum desiccators, where air bubbles were removed. Finally, the form was closed and placed into a drying oven, where the material was cured at 100 °C for 2 h. 

### 3.6. Preparation of Modified CNT–DBSA/Silicone Composites by Mechanical Mixing

The composites of the third set with the concentration of DBSA-modified CNTs ranging from 0 to 3.5 vol.% were prepared in the same manner as the composites in Section 3.4. 

### 3.7. Preparation of Modified CNT–DBSA/Silicone Composites by Sonication

The composites of the fourth set with the concentration of DBSA-modified CNTs ranging from 0 to 6 vol.% were prepared in the same manner as the composites in Section 3.5.

### 3.8. Transmission Electron Microscopy

The morphology of the samples was investigated by a Tesla BM 500 transmission electron microscope at 90 kV (Tesla, Prague, Czech Republic). We placed a drop of CNT suspension in water or in a surfactant solution (DBSA, CTAB, or mixture of surfactants) on the electron microscope grid and, after evaporation of water at room temperature, investigated the sample by an electron microscope.

### 3.9. Scanning Electron Microscopy

Scanning electron microscopy (SEM) analysis was carried out by a Zeiss Gemini Supra microscope (Oberkochen, Germany) on thin 500 μm-thick chips cut at room temperature. The structure of a silicone composite was observed on a chip by EBL (Electron Beam Lithography) lithographer (e-Line RAITH GmbH, Dortmund, Germany). The samples were observed without gold sputtering of surface. 

### 3.10. DC Conductivity

The current–voltage characteristics were measured by a Keithley 6517A programmable electrometer (Keithley, Cleveland, OH, USA), which was also used as a DC power source. The Van der Pauw four-point method [32] was used for measuring samples with conductivity higher than 10^−1^ S·m^−1^. The main advantage of the four-probe method is the elimination of contact resistance. We calculated the electric conductivity *σ_DC_* by the equation:

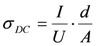
(2)
where *I* is electric current, *U* is voltage, *A* is the area of electrodes, and *d* is the width of the sample. The average values 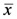 and standard deviations *δ* were obtained from five measurements. All the properties were measured at room temperature (22–25 °C).

### 3.11. Dielectric Properties and AC conductivity

The dielectric properties of CNT–silicone composites were measured by a Hioki 3522 LCR bridge in the frequency range 100 Hz–100 kHz and by an Agilent 4991A (Agilent, Santa Clara, CA, USA) impedance material analyser in the range from 1 MHz to 3 GHz. The values of the impedance *Z*, capacity *C*, conductivity *G* and the loss factor tan*δ* were measured by a Hioki 3522 LCR bridge (Hioky, Nagano, Japan). Then the conductivity *σ_AC_* was calculated by Equation (3), the real part of permittivity *ε'* was calculated by Equation (4), and the imaginary part of permittivity *ε"*, by Equation (5):

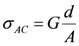
(3)

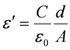
(4)

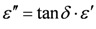
(5)


The *ε'*, *ε"* and tan*δ* were measured by an Agilent 4992A impedance material analyzer, and the *σ_AC_* is calculated by the Equation (6):

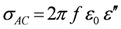
(6)
where *f* is frequency and *ε_0_* is the permittivity of vacuum.

### 3.12. Thermal Conductivity

For measuring the thermal conductivity by a non-stationary method, one uses an instrument described in [33]. The instrument is schematically shown in [34]. This instrument is usually applied for measuring the thermal conductivity of thin sheets or slabs of various plastics rubber and leather.

**Figure 12:**
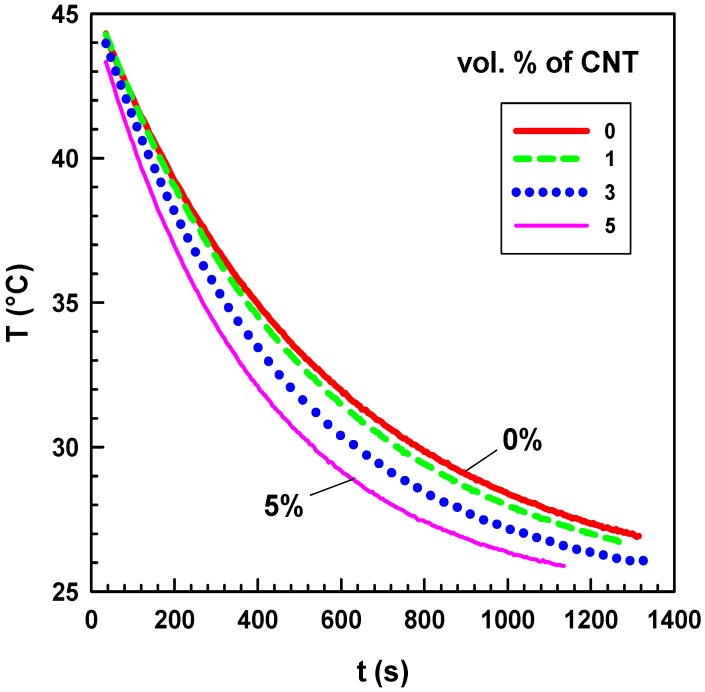
Temperature as a function of time for CNT/silicone composites with various CNT contents.

The measurement principle is as follows: initially, the central brass cylinder (CBC) with diameter of 5 cm is annealed to reach *T*_2_ = 45 °C with the help of another hollow brass cylinder that is connected to a water thermostat by rubber hoses. The water thermostat’s accuracy was 0.1 °C. Then the 45 °C CBC is quickly removed, and a test sample with diameter of 5 cm and thickness of about 2 mm is placed on the top of the CBC, and another hollow brass cylinder connected to another water thermostat with temperature *T*_1_ = 25 °C is placed on the top of the test sample. Finally, a 100-g weight is placed on the top of the pile. The heat is transferred from the CBC through the sample to the colder brass cylinder (25 °C). The temperature of the CBC rapidly decreases. The temperature of the CBC is measured by a copper–constantan thermocouple connected to an NI USB-9211A data acquisition equipment (Portable USB-Based DAQ for Thermocouples), which is connected to a computer through a USB port. The measurement takes about 25 min. The non-linear analysis (exponential decay) of the temperature versus time curve is performed by SigmaPlot 12 software. Raw measurement data (temperature as a function of time for various nanocomposites) are shown in Figure 12.

The variation of the temperature of the composite with 5 vol.% of CNT is much faster than that of the composite with 1 vol.%. The mathematical model and the details of the thermal conductivity measurement are described elsewhere [34]. The measurement of each sample was repeated five times.

## 4. Conclusions

The modification of CNTs by anionic surfactant (DBSA) and sonication leads to a homogeneous distribution of a filler in a silicone matrix. This is reflected in the increased value of percolation threshold and the decreased values of complex permittivity and thermal conductivity of CNT–DBSA-based composites compared with those of composites filled with neat CNTs. The increased homogeneity of CNT–DBSA-based silicone composites and the separation of CNTs in the silicone matrix can be observed by SEM analysis and the measurement of the electrical and thermal conductivity of the composites. The separation of CNTs decreases the probability of percolation cluster formation, but increases the area of the CNT–polymer matrix interface. The former increases the value of percolation threshold, while the latter is responsible for the decrease of the thermal conductivity due to high phonon scattering on the CNT–polymer matrix interface.
